# Hospital Meetings

**Published:** 1907-03-23

**Authors:** 


### HOSPITAL MEETINGS.

### GREAT NORTHERN CENTRAL HOSPITAL.

The annual meeting took place on Thursday, March 14. The number of patients was larger than in the previous year, the in-patients amounting to 2,301, as against 2,170 in 1905, and the out-patients to 30,236, as against 28,781; but the average of attendances had decreased, owing probably to the improved system of admission. By this system patients were now kept in two observation wards for some time until the authorities were satisfied of their suitability for admission, and by this means many chronic and other unsuitable cases were referred for treatment to other agencies.

#### Hospital Abuse.

The Chairman said that there was no department of a hospital so susceptible of abuse as the out-patient department, and he much regretted that the hospitals of the Metropolis did not adopt a more uniform method of admission. The Great Northern Hospital had always been alive to the dangers of abuse, and was continually devising safeguards. A scheme for the better management of the department had been adopted, whereby it was hoped to exclude patients able to pay and those more suitably treated elsewhere; and to this end a casualty officer and a lady almoner were necessary in the place of the former inquiry officer. The Committee was paying the closest attention to the expenditure of the hospital, but it was impossible to sacrifice efficiency to economy.

#### The Wants of the Hospital.

It was felt that it was highly desirable to establish an electrical department, as at present all the *x*-ray work had to be done outside the hospital at a cost of nearly £200 per annum. There was also much need of a children's ward; but as this would mean an initial expenditure of about £650 and an annual increase of about £600, the Committee did not feel justified in embarking on this. They hoped, by their new plan of calling upon all residents in the neighbourhood to contribute, to gain more local support, as at present out of a daily expenditure of £45 Islington only contributed about £6.

The nine Governors of the Committee of Management were re-elected, with the exception of Sir Benjamin Cohen, Bart., who resigned on account of ill-health. At the suggestion of the Committee of Management, the Council of Governors decided in favour of the election of Lady Governors as members of the Committee of Management, the number of ladies so elected not to exceed three. In accordance with this decision Mrs. Lansdowne Beale, Miss Amy Hill, and Miss David Waterlow were elected.

The meeting expressed its sense of loss by the death of the Hon. R. A. Capel, one of the original trustees, and a member of the Committee of Management since 1871; and also by the death of Baroness Burdett-Coutts, who had helped the hospital so much by her influence.

Annual Meetings.—Home Hospitals' Association (for paying patients), at Fitzroy House, 16, 17, 18 Fitzroy Square, W., on Tuesday, March 26, at 4 p.m.; St. Peter's Hospital, Convent Garden, W.C., at the Hospital, on Tuesday, March 26, at 5 p.m.

The Royal Insurance Company has issued the fourth edition of its neat and handy "Record of Sports" booklet, giving, as clearly and concisely as one could wish for, the records in no less than thirty-five different kinds of sport. All these records are compiled from official sources, and are therefore absolutely accurate. Several pages are devoted to amateur athletic records, a most interesting *résumé* being given of the several championship events. Each section is prefaced by a short explanatory note. In addition, the score of the players at the open golf championships and summaries of international and inter-university (though not of inter-hospital) contests are included. The booklet, which will appeal to every sportsman, whether his interest be limited to croquet or catholic enough to embrace all the varieties of recreation recorded, may be obtained gratis on application to the Manager, Royal Insurance Company, 1 North Street, Liverpool.

